# Strengthening tactical planning and operational frameworks for vector control: the roadmap for malaria elimination in Namibia

**DOI:** 10.1186/s12936-015-0785-1

**Published:** 2015-08-05

**Authors:** Emmanuel Chanda, Birkinesh Ameneshewa, Hans A Angula, Iitula Iitula, Pentrina Uusiku, Desta Trune, Quazi M Islam, John M Govere

**Affiliations:** Kamwala South, Lusaka, Zambia; World Health Organization, Regional Office for Africa, Brazaville, Republic of Congo; Namibia National Vector-borne Diseases Control Programme, Windhoek, Namibia; World Health Organization, Country Office, Windhoek, Namibia; Nelspruit, Mpumalanga South Africa

**Keywords:** Malaria vector control, Integrated vector management, Insecticide resistance management, Strategic planning, Operational frameworks, Vector surveillance, Community involvement, Monitoring and evaluation, Namibia

## Abstract

**Background:**

Namibia has made tremendous gains in malaria control and the epidemiological trend of the disease has changed significantly over the past years. In 2010, the country reoriented from the objective of reducing disease mor bidity and mortality to the goal of achieving malaria elimination by 2020. This manuscript outlines the processes undertaken in strengthening tactical planning and operational frameworks for vector control to facilitate expeditious malaria elimination in Namibia.

**Case description:**

The information sources for this study included all available data and accessible archived documentary records on malaria vector control in Namibia. A methodical assessment of published and unpublished documents was conducted via a literature search of online electronic databases, Google Scholar, PubMed and WHO, using a combination of search terms.

**Discussion and evaluation:**

To attain the goal of elimination in Namibia, systems are being strengthened to identify and clear all infections, and significantly reduce human–mosquito contact. Particularly, consolidating vector control for reducing transmission at the identified malaria foci will be critical for accelerated malaria elimination. Thus, guarding against potential challenges and the need for evidence-based and sustainable vector control instigated the strengthening of strategic frameworks by: adopting the integrated vector management (IVM) strategy; initiating implementation of the global plan for insecticide resistance management (GPIRM); intensifying malaria vector surveillance; improving data collection and reporting systems on DDT; updating the indoor residual spraying (IRS) data collection and reporting tool; and, improving geographical reconnaissance using geographical information system-based satellite imagery.

**Conclusions:**

Universal coverage with IRS and long-lasting insecticidal nets, supplemented by larval source management in the context of IVM and guided by vector surveillance coupled with rational operationalization of the GPIRM, will enable expeditious attainment of elimination in Namibia. However, national capacity to plan, implement, monitor and evaluate interventions will require adequate and sustained support for technical, physical infrastructure, and human and financial resources for entomology and vector control operations.

## Background

Malaria remains the most important vector-borne disease globally and continues to severely undermine the socio-economic growth in sub-Saharan Africa where it exacts its greatest toll, particularly in children and pregnant women [1, 2]. In 2012, there were an estimated 207 million malaria cases and 627,000 deaths globally with almost 84% of the cases and 92% of the deaths reported from sub-Saharan Africa [3]. The post-2015 global technical strategy for malaria supports countries in reducing the disease burden and accelerating progress towards elimination [4]. The strategy accentuates the strategic direction, sets milestones and targets to 2030, and provides a framework for countries to adapt to the national strategic plans. While several documents to guide the transition from malaria control to elimination exist [5, 6], endemic countries have faced a multiplicity of challenges on their path to malaria elimination [7].

To mitigate vector control constraints, the World Health Organization (WHO) recommends adoption and implementation of integrated vector management (IVM) along the five key strategic elements as a platform for effective vector control [8] and the global plan for insecticide resistance management (GPIRM) along its five pillars as a means of preventing the development and spread of resistance [9]. Furthermore, guidelines for vector surveillance to facilitate entomological data collection [10], guidance documents on strengthening of reporting systems on dichloro-diphenyl-trichloroethane (DDT) in relation to disease vector control [11] and training modules for vector control and entomology [12] have been developed. To facilitate efficient and timely implementation, the WHO has developed operational plans for IVM, a framework for the GPIRM [13] and operational manuals for indoor residual spraying (IRS) [14], larval source management (LSM) [15], and long-lasting insecticidal nets (LLINs) [16].

In 2009, the elimination eight (E8) initiative was established consisting of countries with potential of malaria elimination: Botswana, Namibia, South Africa and Swaziland including their neighbours Angola, Mozambique, Zambia and Zimbabwe [17]. The government of Namibia launched a malaria elimination campaign in 2010 and reoriented from the objective of reducing disease morbidity and mortality to the goal of achieving elimination by 2020 [17]. Accordingly, systems are being strengthened to identify and clear all infections, and significantly reduce human–mosquito contact [18]. Namibia envisions progressing towards elimination through a phased approach with some districts targeting identified foci to interrupt transmission and others consolidating control before entering the pre-elimination phase [18].

Adaptation of interventions both to the biology and behaviour of local vector species counting the geography and epidemiology of the targeted malaria foci is imperative in the preparatory phase [10]. As Namibia transitions towards elimination, strengthening vector control as a vital attack weapon of reducing transmission will be critical. Thus, guarding against potential challenges and the need for evidence-based and sustainable vector control prompted the consolidation of strategic frameworks by: adopting the IVM strategy [18]; initiating implementation of the GPIRM [19]; intensifying malaria vector surveillance [20]; strengthening data collection and reporting systems on DDT [21]; updating the IRS data collection and reporting tool; and, improving geographical reconnaissance through geographical information system (GIS)-based satellite imagery [21, 22]. This manuscript outlines the processes undertaken in strengthening tactical planning and operational frameworks for vector control to facilitate informed decision-making for expeditious attainment of malaria elimination in Namibia.

## Case description

The National Vector-borne Disease Control Progra mme (NVDCP) in Namibia with its endeavour to strengthen tactical planning and implementation frameworks for vector control to accelerate achievement of malaria elimination was the ‘case’ for this study. WHO Namibia in collaboration with the African Regional Office (AFRO) developed the terms of reference for the study to: harmonize and build consensus on DDT reporting; train staff in vector resistance monitoring techniques; develop guidelines for conducting vector surveillance for regional entomological teams; develop a national insecticide resistance monitoring plan, and to review and update the national IRS data collection and reporting tool. Information sources for this study included all available data and accessible archived documentary records on malaria vector control in Namibia. A methodical assessment of published and unpublished documents was conducted via a literature search of online electronic databases, Google Scholar, Pub Med and WHO, using a combination of search terms. Additional, non-peer reviewed literature was examined for information related to the subject.

## Malaria epidemiology

The epidemiological trend of malaria disease in Namibia has changed significantly over the past years [17]. There has been a sustained impact on malaria disease burden, evidenced through the reduction in the morbidity and mortality of over 99.3% between 2000 and 2012 [17]. The sharpest decline occurred from 2006 following the introduction of artemether–lumefantrine and improved IRS coverage. These achievements paved the way for Namibia to be among four countries in Southern Africa currently having potential to eliminate malaria.

## The temporal and spatial distribution of malaria

**Fig. 1:**
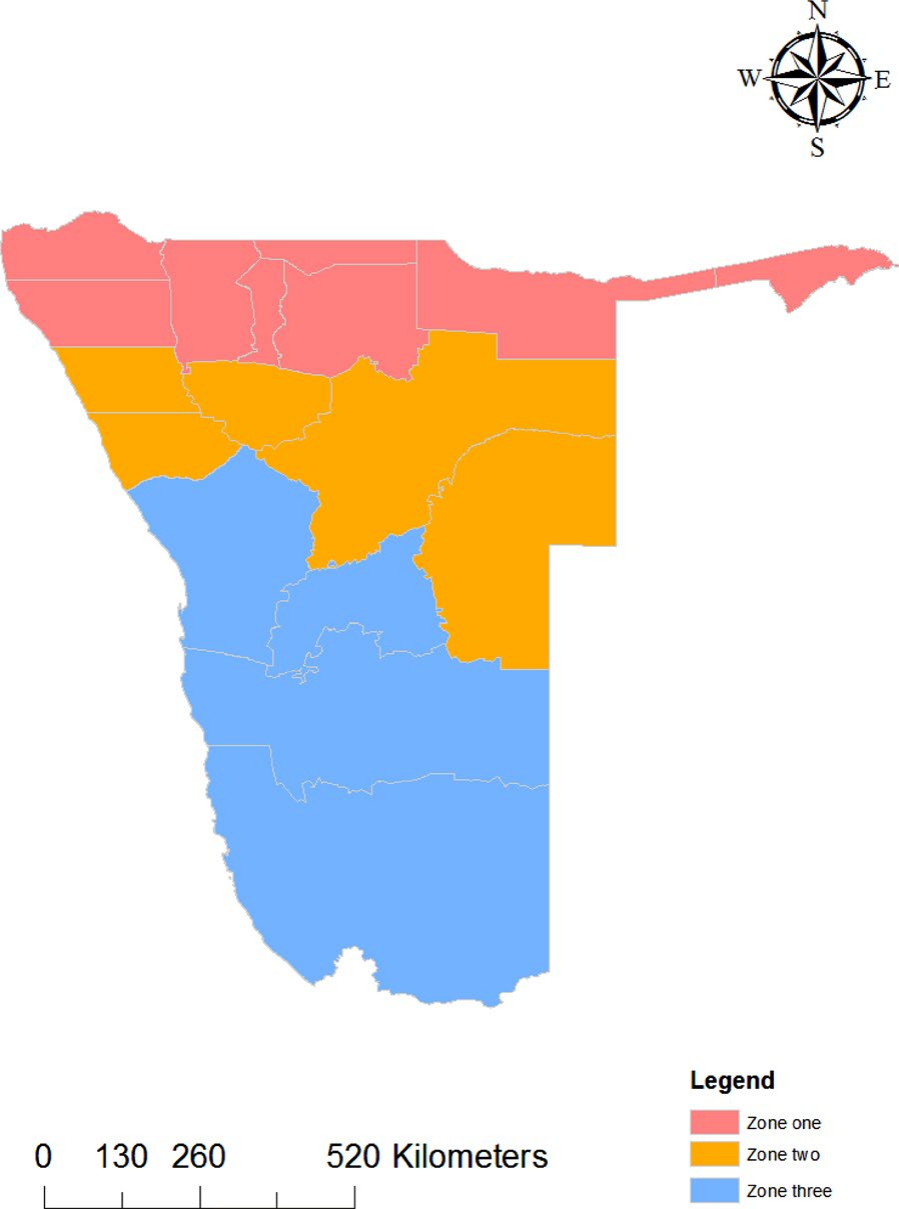
Malaria stratification i n Namibia 2013:* zone 1* moderate transmission risk,* zone 2* low transmission risk,* zone 3* malaria risk “free”.

Though malaria is endemic in nine out of the 14 regions of Namibia, the intensity of transmission is generally low [17]. The northern regions constituting 8% of the country’s total surface area are characterized by an ecosystem of high temperature, rainfall and humidity. Malaria occurs seasonally with periodic focal outbreaks in the northwest and high perennial transmission in the northeast, primarily influenced by rainfall patterns [18]. Presently, malaria receptivity is sustained in Kunene, Omusati, and Ohangwena regions. Endemicity is highest in Kavango and Zambezi regions. The arid coastal and southern regions of Erongo, Hardap, Khomas and Karas are considered free from the disease [23]. Malaria transmission in the country has been stratified into three zones, i.e. Zone 1 (moderate transmission), Zone 2 (low transmission) and Zone 3 (Malaria risk free) (see Fig. 1). About 70% of the population lives in areas where there is some risk of malaria transmission [18].

## Malaria parasites and vectors

In Namibia *Plasmodium falciparum* accounts for 97% of malaria cases, the remaining 3% is due to *Plasmodiumvivax* [17]. Historical entomological baseline data for IRS collected in 1965 demonstrate existence of *Anopheles gambiae* s.s.*, Anopheles funestus* and *Anopheles arabiensis* in Namibia [24]. However, recent studies have consistently shown the absence of the endophilic *An. gambiae* and *An. funestus* due to effective indoor-targeted vector control, leaving *An. arabiensis* as the solitary principal malaria vector [25]. *Anopheles arabiensis*, a vector with facultative resting and feeding behavioural attributes, and predominates in arid areas with low rainfall, has the widest distribution in the northern region of the country [26]. This vector species breeds in water bodies that are temporary and semi-permanent in nature, rests both indoors and outdoors and tends to feed at night, biting humans indoors as well as cattle outdoors [27]. This diversity in resting and feeding behaviour can render *An. arabiensis* less amenable to control by conventional control tools [23].

## Insecticide susceptibility and cone bioassays

To ensure that transmission-interrupting tools remain effective, and their choice is evidence based, insecticide surveillance and monitoring, and contact bioassays are essential [28]. In Namibia, insecticide susceptibility profiles of local malaria vectors, quality of spraying and persistence of bio-efficacy of an organophosphate (DDT) and pyrethroid (deltamethrin) have been assessed using WHO standard protocols from 2009 to 2013 in all malarious regions of the country [29]. In 2009, total susceptibility (100% mortality) in *An. arabiensis* was demonstrated to 4% DDT and 0.05% deltamethrin insecticides currently used for IRS and insecticide-treated nets (ITNs) in the country. In 2010 and 2013, the results for the two insecticides still remained the same in *An. gambiae* s.l. WHO contact bioassay to assess and compare the residual efficacy of DDT 75% WP at 2 g/m^2^ and K’othrine 50 WP and WG 250 at 20 mg/m^2^ for IRS on cement and mud-rendered walls inside houses consistently indicated complete killing effect post 24 h exposure during the entire study period [29].

## Malaria vector control

The NVBDP is responsible for developing the overall operational design, policies and strategies, and coordination and management of all vector control programmes in Namibia. Transmission-reducing interventions (LLINs, IRS and LSM) are implemented as the main malaria vector control tools and are recorded at district level by the Ministry of Health and Social Services in collaboration with community health workers [30].

## Indoor residual spraying

**Fig. 2:**
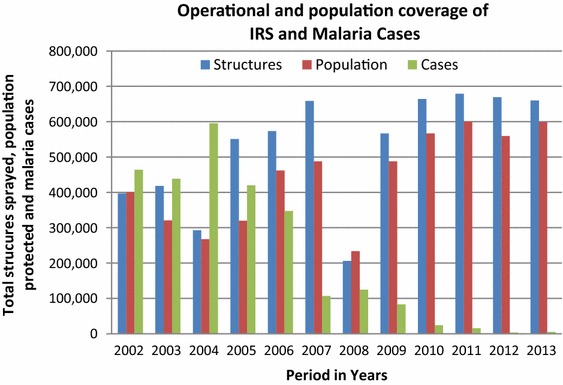
Operational and population coverage of IRS and malaria cases in Namibia 2002–2013.

Intra-domiciliary spraying using DDT dates back to 1965 in northern Namibia [31–33]. More than 1,600 tonnes of DDT was used to spray about 12.4 million housing structures in Ovambo, Kavango and Caprivi from 1966 to 1979 [23]. Bendiocarb and deltamethrin were introduced in 1980 and 1990s respectively. Currently, DDT is mainly used on traditional structures and deltamethrin is used on modern cement block structures. Between 2001 and 2013, over 742 tonnes of DDT and 32.6 tonnes of deltamethrin were used to spray in excess of 6.7 million structures in all malaria endemic regions (Fig. 2). The annual spraying cycle falls between September and December, timed to start just before the onset of the rainy season. IRS is coordinated and carried out by EHOs at regional and district levels who also supervise spray teams comprised of temporary labourers. Annual IRS operational coverage remains above 80% since 2005, except in 2008 when the coverage dropped to 38% due to procurement challenges [27]. The national programme also conducts supervisory visits during trainings and in the field. For quality control, bioassay testing is conducted annually to monitor the correct application of IRS.

## Insecticide-treated nets

Distribution of ITNs to women only began in 1993 in northern Namibia. In 2002, targeting of ITNs to only children <5 years of age and pregnant women was commenced. Between 2002 and 2004, about 52,000 ITNs were distributed at no cost [17]. In 2005 a policy change instituted broader targeting of at-risk groups, including children under 5 years of age and pregnant women with LLINs complimented by mass distribution. LLIN distribution was augmented in 2008 to compensate for lower IRS coverage [18]. In 2009, the distribution policy further shifted towards universal coverage with one LLIN per two people in moderate transmission areas. In the same year, 53% of households had at least one LLIN and usage among children <5 years and pregnant women was 34.0 and 25.9%, respectively [34]. However, since 2010, distribution of LLINs targeted the general population to accelerate universal coverage. From 2005 to 2011, over 625,000 LLINs were distributed at health facilities, outreach sites, antenatal clinics, and via mass campaigns to villages [27]. In 2012, the NVDCP set a new goal to achieve 95% LLIN coverage of the entire population, shifting from just vulnerable populations to all those living in regions with any risk of malaria transmission by 2014. In 2013, a mass distribution of 87,900 LLINs was targeted to villages with the highest malaria caseloads in Zambezi, Kavango,and Omusati [27].

## Larval source management

Targeted winter larviciding has been used as an effective intervention for reducing malaria transmission in the southern part of Africa. The NVBDP has been applying winter larviciding during the dry season, which is conducted at limited vector breeding sites in malaria endemic regions [30].

## Rationale for consolidating the strategic framework

In Namibia, malaria vector control efforts have been characterized by several technical, institutional, infrastructural and programmatic constraints (Table 1): insecticide resistance monitoring in local malaria vectors has been inconsistent and limited to only two insecticides DDT and deltamethrin. While insecticide resistance has not been detected, the skills to manage it are still scarce. Contact bioassays to assess both the quality and residual efficacy of the sprayed insecticides have only been conducted once annually instead of monthly. Malaria vector surveillance has been very minimal to substantiate the claim that presently *An. arabiensis* remains the solitary vector in Namibia. No county-specific rational vector surveillance guidelines existed and entomological monitoring has been conducted on an ad hoc basis. Furthermore, the country lacks adequate entomological laboratory and insectary capacity. Vector control and entomological data collection, management and dissemination have been hampered by a lack of institutionalized databases and repository including limited coordination and collaboration. The IVM strategy has not been fully harnessed for effective vector control resulting in minimal or lack of intersectoral collaboration between agriculture, health and environmental programmes, as well as weak cross-border coordination and collaboration with Angola and Zambia. The situation is further compounded by minimal documentation and reporting for DDT. Supplementary interventions such as LSM have been inconsistently conducted in Namibia. Operational experience with IRM to facilitate systematic IRM deployment is relatively minimal together with implementation of new policies in the context of the GPIRM to optimize their impact. Addressing these challenges would enable evidence-based deployment and optimal assessment of interventions to inform future policies for effective operational vector control.

**Table 1:** Challenges and risks encountered in vector control and recommendations for improvement in Namibia

Challenges and risks encountered	Recommendations for improvement
Minimal data is available on susceptibility testing in all malarious regions, small number of sentinel sites and limited mapping data of malaria vector species	Establishing an efficient system of sentinel sites to support monitoring of vector density, infectivity, bionomics and insecticide resistance to guide informed decision-making
The possibility of change in behaviour of *An. arabiensis* from indoor resting to outdoor resting in response to indoor spraying, and a change in daily pattern of biting and host choice in response to ITNs or infectivity rates	Integrated strategies with actions aiming to reduce the selection pressure thus preventing or delaying resistance development, and aiming to reduce dependency on chemical insecticides for vector control
Inconsistent community sensitization and low compliance with existing interventions and insufficient community ownership and involvement in malaria vector control activities	Improve information, education and behavioural change communication on vector control interventions and reinforce community engagement and empowerment for participation to successfully implement malaria vector control
Limited support institutions with requisite infrastructure to support entomological research; minimal research and academic institutions to build capacity of sufficient and technically sound personnel and the likely hold of resistance development in major malaria vectors to insecticides	Establish a multi-disciplinary national IRM decision-making body coordinated by the NVDCP, and the inherent partner commitment and collaboration required to strengthen entomological laboratory and operations research and implement rational IRM strategies
Absence of comprehensive malaria transmission data and minimal utilization of existing entomological data for decision making for vector control	Improve vector surveillance, collect entomological data and strengthen operational research and monitoring and evaluation to guide the scale up of interventions
Lack of stakeholders alliance for effective reporting of insecticides used for diseases vector control particularly DDT	Establish stakeholders’ alliance and develop agreed upon roles and responsibilities and standardized format for compilation and reporting on DDT to the focal person of the Secretariat of the Stockholm Convention
Non existence of data management for quantities and total cost of insecticide used and cost of spraying cycle for each year to facilitate decision making for vector control	Establish and operationalize a comprehensive entomological database at national level and incorporate record keeping and reporting in training curriculum and courses on IRS and other vector control operations
Weak infrastructure (sentinel sites and laboratory and insectary facilities), technical and human resource capacity for entomology laboratory at the established sentinel sites	Seek technical assistance to support entomological surveillance, particularly for capacity building in vector-bionomics and insecticide resistance to operationalize the implementation of the GPIRM
Human, financial and logistic resources/capacity to implement, monitor and evaluate IVM is still minimal	In-service training for the entomological laboratory staff in malaria entomology and vector control should be considered will be critical for sustained evidence decision making in the regions

## Opportunities for strengthening vector control

In 2014, Namibia sought for technical support from WHO/AFRO to develop and update the existing vector control strategic and operational frameworks.

## Adopting and implementing the IVM strategy

The national IVM guidelines have been developed to facilitate evidence-based deployment of tools and thus strengthen vector control [18]. These guidelines provide information on the components of the Global strategic framework for IVM and present technical contents including planning, implementation, supervision, monitoring and evaluation of IRS, ITNs and LSM. The IVM strategy is being developed to give the tactical direction for effective deployment of key vector control interventions along the five key elements of the approach (Table 2) and to align them with changing epidemiology of the disease in Namibia. It outlines strategic interventions and activities, Insecticide resistance management (IRM), monitoring and evaluation and operational research, programme management, budget and funding and an implementation plan. The strategy is expected to reduce the risk for transmission, reduce the disease burden, improve the cost-effectiveness of operations, improve ecological soundness and be sustainable.

**Table 2:** Key elements of an integrated vector management (IVM) strategy

No.	Element	Description
1	Advocacy, social mobilization and legislation	Promotion and embedding of IVM principles in designing policies in all relevant agencies, organizations and civil society; establishment or strengthening of regulatory and legislative controls for public health; empowerment of communities
2	Collaboration within the health sector and with other sectors	Consideration of all options for collaboration within and between public and private sectors; application of the principles of subsidiarity in planning and decision-making; strengthening channels of communication among policy-makers, vector-borne disease programme managers and other IVM partners
3	Integrated approach	Ensure rational use of available resources by addressing several diseases, integrating non-chemical and chemical vector control methods and integrating with other disease control methods
4	Evidence-based decision-making	Adaptation of strategies and interventions to local ecology, epidemiology and resources, guided by operational research and subject to routine monitoring and evaluation
5	Capacity-building	Provision of the essential material infrastructure, financial resources and human resources at national and local level to manage IVM strategies on the basis of a situational analysis

## Initiating the implementation of the GPIRM

Transitioning to strategic IRM required elaboration of a resistance management and monitoring plan based on a thorough situation analysis of insecticides registered for public health and agricultural use, main vector species and their insecticide susceptibility status, data management, dissemination and mapping, vector control interventions, evidence and knowledge gaps and constraints, risks and mitigating measures [35]. The objectives of the IRM plan is to: (1) monitor the spatiotemporal vector resistance status to all four classes of insecticides; (2) determine the underlying resistance mechanisms and, (3) establish a data base to facilitate rational IRM strategies. It has an implementation framework comprising a multidisciplinary national IRM decision-making body. Multiple sentinel sites for routine resistance surveillance and monitoring have been established and strengthened in three malarious zones of Namibia. Data collected annually will be harnessed for streamlining vector control within the context of the IVM strategy. The framework also include, interpretation of test results and policy implications, human resources, procurement and supplies, regulatory requirements and procedures, budget and potential sources of funding [35]. Mechanisms to improve reporting on insecticide resistance have been established.

## Consolidating malaria vector surveillance

As Namibia transitions to malaria elimination, vector surveillance will be an integral aspect of the IVM strategy in two ways: (1) to provide evidence for decision-making in IVM and (2) for evaluating a programme’s impact on vector populations. It will also be used for monitoring and evaluation where the surveillance sites are located in or near the implementation settings. These investigations would provide information on malaria vector species composition, their distribution, population density, feeding and resting behaviour, infectivity rate, longevity of vectors, seasonal activities, larval habitats, susceptibility to insecticides, and quality and residual effect of insecticides used for malaria control [20]. Because of a long sustained IRS programme in Namibia which reduced vector populations to very low levels, some of the indicators mentioned here may be difficult to measure. In this regard, vector surveillance will support case detection and stratification of malaria foci using both epidemiology and entomology data for focal application of IRS in areas where malaria cases are very few to justify blanket spraying, which is important in malaria elimination programmes.

Country-specific vector surveillance guidelines have been developed and include: entomological field and laboratory techniques, WHO contact and susceptibility bioassays, mosquito rearing in the insectary, organization of entomological teams, geographical information systems (GIS) and supervision of entomological teams and operations [20]. Namibia is implementing an elimination strategy as such, stratification (mapping) of active, passive, new foci becomes imperative. Malaria is generally heterogeneous in distribution except in areas along the Kavango river, where disease distribution remains generally homogenous. In this regard spot checks to support surveillance at sentinel sites with active vector surveys conducted together with case detection teams are recommend and investigations will be carried out regularly at fixed locations to: (1) reduce natural variation, costs and labour intensity; (2) to increase the usefulness of timely collected data from surveillance in decision-making, and (3) to maximize the use of available resources. Thus sentinel sites are being strengthened and training of national staff in basic entomological monitoring, field and laboratory techniques including insectary management has been embarked upon in Namibia [20]. While only one insectary exists in Namibia, it has been characterized by a long state of inactivity.

## Strengthening of reporting systems on DDT

Namibia was among the first countries to ratify the Stockholm Convention but delayed the development of the National Implementation Plan and could not establish mechanisms for reporting to the secretariat of the Stockholm convention. To facilitate expeditious implementation of planned activities of the project on “establishment of efficient and effective data collection and reporting procedures for evaluating the continued need of DDT for disease vector control” [36], a workshop on harmonization and consensus building on DDT reporting and reducing the reliance on DDT use for malaria vector control was held [21]: (1) a common understanding on the need for the country to build the capacity and system for effective documentation and reporting of DDT and other insecticides for diseases vector control was created; (2) effective stakeholder alliance terms of references, i.e., roles and responsibilities of intersectoral alliances (NVDCP and the persistent organic pollutants focal point in the reporting of DDT were established; (3) standardized format for compilation and reporting on DDT to the focal person of the secretariat of the Stockholm Convention was adopted and; (4) environmental health practitioners were trained in vector resistance monitoring techniques and data collection and reporting procedures of DDT using WHO guidelines. Sources of information and data, or specialized agency required for DDT reporting and the focal persons were identified and annual coordination meetings scheduled; plans to incorporate record keeping and reporting in training curriculum and courses on IRS and other vector control operations are underway; efforts to establish an up to date data base for annual data entries at NVDCP have been embarked upon, and modalities for timely questionnaire completion and submission of the report have been established [21].

## Updating the national IRS data collection and reporting tool

A methodical review of national published and unpublished data from all relevant documents and staff in the MoHSS and involved partner institutions was collected and collated. The national IRS information/data collection and reporting tool has been updated in line with the regional and global relevant documents, and compiled in the WHO standard format for IRS [22]. The data recorded include; IRS policy/strategy in the reported years outlining the objective for spraying (malaria elimination, seasonal or perennial malaria control, epidemic response) and specify whether targeted or blanket spraying. Thorough details of national and district coverage of IRS (population at risk of malaria, population at risk of epidemics, operational coverage of IRS-structures targeted, structures sprayed, percentage coverage and refusal rate—and population coverage of IRS—population targeted, population protected and percentage coverage) and specifying the implementers (NMCP or partners/contractors). It also indicates insecticides used (products/class, formulation, dose (mg/m^2^), quantities (Kg/Li), cost of insecticides, total cost of IRS and source of funds). It also includes IRS implementation (who conducted IRS), supervision (checklist and reports) and quality monitoring using cone bioassay including vector surveillance. The tool also incorporates current pesticide management capacity of the IRS programme (transport and storage, safe handling and waste management) [22].

## Improving geographical reconnaissance

Following many years of IRS in Namibia, it would be expected that the programme has a clear knowledge of number and location of structures. However, the IRS programme in Namibia experiences various challenges, including: (1) planning—number and type of structures, location of structures and spray operators needed; (2) targeting—using incidence from previous year only can leave out receptive areas that have had cases in recent past; (3) operations—inadequate number of supervisors, lack of geographical reconnaissance data/not available in field leads to inefficient movements, and managers/supervisors have no access to real-time data; (4) logistics—transportation, spray pumps, paying spray operators; (5) advocacy, and (6) monitoring and evaluation—inaccurate coverage data and difficulty in mapping of sprayed/missed areas). In this regard, conducting a thorough and timely GR supported by GIS-based satellite imagery is critical to circumvent these constraints [21]. This will utilize a new and efficient approach using satellite imagery to enumerate each household in the country and will improve IRS planning, operations, and monitoring. Moreover, the approach is cheaper, faster, requires fewer human and financial resources and ensures 100% coverage of households/villages with satellite view. This will also facilitate targeting and prioritization of eligible spray areas together with operations and real time monitoring of spray coverage.

## Discussion and evaluation

Further to the evidence-based deployment of effective and proven malaria control interventions by the NVDCP, tremendous change in the epidemiology of the disease has been experienced across Namibia [30]. The country is at an exciting turning point with its elimination potential. However, there is an inherent risk of complacency and lack of motivation that needs to be guarded against, as historically evidenced in other countries who have reached elimination [17]. If efforts are not maintained, a resurgence of malaria could easily occur, threatening the progress and gains made to date. A sustained political and financial commitment will be necessary for Namibia to remain malaria free once elimination is achieved. While case management and surveillance are key, strengthened vector control will be critical during the pre-elimination phase in Namibia [18].

The IVM strategy defined by the WHO as “a rational decision-making process to optimize the use of resources for vector control” is a combination of proven malaria vector control methods based on the knowledge of local vector biology and ecology of the disease transmission [8]. This can be a singular intervention, but is usually a combination of multiple interventions in a synergistic approach. Integrated vector control methods include IRS, distribution of LLINs, larviciding and personal protection methods both synthetic and traditional. IRS and LLIN distribution are the hallmark vector control interventions in Namibia [18]. However, these should be implemented based on annual review of malaria vector bionomics, susceptibility studies and disease transmission. In this regard, the NVBCP is considering adoption of contemporary innovative vector control strategies including eave tubes and Entomopathogenic bacteria traps, durable wall linings including personal protection to combat outdoor transmission through Entomopathogenic fungus-impregnated targets, attractive toxic sugar baits and spatial repellents.

The fact that insecticide resistance selection pressure is driven primarily by gene flow, agricultural and public health use of insecticides as well as cross resistance has got implications for malaria vector control in Namibia. While no insecticide resistance in malaria vectors has been reported so far in Namibia, resistance has been detected in all the neighbouring countries except Botswana. In Angola, knockdown resistance (*kdr*) west—the L1014F mutation—has been found in *An. gambiae* M-form individuals [37]. In Zambia *kdr* west (1014F) and east (1014S) alleles to pyrethroids and DDT, and over expression of cytochrome P450s in pyrethroid resistance and glutathione S-transferases (GSTs) in DDT resistance has been reported in *An. gambiae* s.s, including over expression of GSTs in pyrethroid resistance and carbamate resistance in *An. funestus* s.s. [38, 39]. In Zimbabwe, *An. arabiensis* resistance to both DDT and permethrin has been reported in Gokwe District with high activity levels of P450s, GSTs and general esterase activity detected [40]. In South Africa, high resistance to pyrethroids in *An. arabiensis* has been demonstrated [41], including P450-based metabolic resistance to DDT in *An. gambiae* s.l. [42] and P450 monooxygenase and/or GST-mediated pyrethroid resistance in *An. funestus* [43]. This situation highlights the extent of the resistance problem in southern Africa and necessitates frequent and extensive resistance monitoring in well chosen sentinel sites across Namibia and an urgent need for pre-emptive resistance management strategies preferably annual rotations of insecticides.

In an elimination setting, quality surveillance data is crucial in informing and guiding the targeting of interventions. Though Namibia is transitioning towards malaria elimination, there are still very limited data available on the spatiotemporal bionomics and insecticide resistance status of malaria vector species to guide targeted and effective control, including monitoring of potential vectors and the role they could play in disease transmission. To ascribe the impact to vector control activities, such information on vector attributes must be known and data needs to be collected in a timely manner to serves as an evidence base for decision-making in IVM. However, some of these activities may require special expertise and equipment. For the purpose of evaluating local effects of IVM, new sentinel sites should be selected in the intervention and control areas [20]. While vector surveillance systems are often concentrated on one disease, implementation of an IVM strategy will enable vector surveillance to cover the vectors of other vector-borne diseases, thus improving the efficiency of resource use in Namibia. The NVDCP should establish data quality self-assessment (DQS) system to ensure accuracy, timeliness and completeness [17]. The DQS should consist of tools that are designed for programme staff at the national, regional and district levels. It helps to evaluate different aspects of the programme monitoring system at region and district levels in order to determine the quality of reported data and address the identified gaps.

While the deployment of an IVM based vector control strategy will facilitate heightened advocacy and resource mobilization [44], the current malaria vector control efforts are characterized by apparent gaps in entomological evidence: key informational gaps that need to be addressed in the short term include; frequent and extensive susceptibility testing, increasing the number of sentinel sites and mapping of malaria vector species. Medium and longer-term knowledge gaps include; vector biting and resting behaviour, host choice or infectivity rates [35]. Short-term action will be to support monitoring of vector density, infectivity, bionomics and quantifying insecticide resistance to guide informed decision-making and to determine the impact of resistance. Long-term solutions will involve establishing a rational IRM plan to reduce the selection pressure thus preventing or delaying resistance development, and aiming to reduce dependency on chemical insecticides for vector control [35].

In Namibia, efficient and effective data collection and reporting procedures for evaluating the continued need of DDT for disease vector control have been successfully established [21]. Potential stakeholders alliance for effective reporting of insecticides used for diseases vector control, particularly DDT, include the Ministry of Environment and the Ministry of Agriculture, water and forestry [21]. The NVDCP should consistently convene annual stakeholders alliance meetings for effective reporting of public health insecticides principally DDT. Strengthened coordination of the stakeholder alliance to facilitate timely and quality compilation and reporting on DDT based on the standardized format to the VBDCP and to the focal person of the Secretariat of the Stockholm Convention should be prioritized. This will enable the conference of parties of the Stockholm Convention, in consultation with the WHO to evaluate the continued need for DDT [11].

With the malaria incidence declining to low levels in Namibia, and the country setting 2020 as the target for malaria elimination, many of the residual malaria cases occur in the border regions with Angola. Presently, transmission is sustained in Kunene, Omusati, and Ohangwena regions constituting the Trans-Kunene Malaria Initiative [45, 46]. Human population movement has been identified as one of the major obstacles to malaria elimination [23]. It is possible that malaria transmission is associated with cross border movements of people between Angola and Namibia. Moreover, the northern parts are wetter and the potential of sustaining the presence of additional Afro-Tropical malaria vectors is high. Given the variable behaviour of *An. arabiensis*, in areas where the mainstay for vector control has been IRS with DDT since the 1960’s, reassessment of the vector species composition and its resting and biting behaviour are important [47]. It is imperative to further explore the presence of *An. funestus* along the border of Kavango and Angola and that of Caprivi and Zambia. The possibility of re-introduction from Angola and Zambia cannot be completely ruled out thus calling for closer coordination of malaria activities through the Trans-Zambezi Malaria Initiative. However, strengthened collaboration and involvement of high level decision makers IN cross-border meetings between the countries will be key and would stimulate and ascertain implementation of recommendations from such meetings.

The mandate of informing, educating and mobilizing the communities on the malaria elimination goals in Namibia rests with the NVDCP. This requires unremitting and well-coordinated information, education and behaviour change communication to promote knowledge and awareness on malaria, create a sense of ownership and guiding all levels of society to achieve malaria elimination. Equally, strategic and effective advocacy to mobilize domestic (public and private) support will be critical for the sustainability of the malaria elimination campaign. To achieve this, the NVDCP should; (1) coordinate the development of all malaria IEC and advocacy material and ensure the harmonization of its content in collaboration with partners, and (2) advocate for increased and continued political, financial, and technical support and mobilize all stakeholders and partners in its efforts to eliminate malaria [17].

The requisite technical and infrastructure capacity for entomological capacity in Namibia remains minimal or nonexistent at all levels of service delivery i.e. national, regional and local levels. This nominal local science capacity evidenced by the very few publications by in-country scientists remains a major challenge for achieving elimination. Cognizant of the fact that building this capacity will take a long time, deliberate efforts to establish strategies to strengthen this aspect of the NVBDP will be critical for expeditious elimination of malaria. As such, networks for empirical and operations research including malaria vector species, bionomics and insecticide resistance monitoring and management should be established and operationalized. This could be achieved through well coordinated collaboration with research and academia institutions i.e. national universities, as well as by creating an enabling environment for external institutions to develop interest in entomological research in the country.

Namibia has implemented a very successful malaria vector control programme using IRS, LLINs and larval control in the context of IVM. The use of other personal protective interventions is being encouraged by the NVDCP and updated malaria vector control policies and guidelines are in place. The success of the malaria control in Namibia has been a function of total government political commitment and deployment of effective control strategies that is decentralized to district level with a strong partnership support and collaboration including the communities. However, the programme has been grappling with a number of challenges in the implementation of interventions including data collection and reporting, technical capacity due to shortage of staff, operational budgets and other requisite resources. As the country re-orients towards malaria elimination, it is imperative that vector surveillance, data collection and reporting, technical and operational constraints are circumvented within the programme. To expedite the transition towards elimination an emergency mode should be adopted by the control programme.

## Conclusions and way forward

Universal coverage with IRS and LLINs, supplemented by LSM in the context of IVM, guided by vector surveillance coupled with rational operationalization of the GPIRM, will enable Namibia to expeditiously attain the goal of elimination. However, national capacity to plan, implement, monitor and evaluate different vector control interventions will require allocation of adequate and sustained support for technical, physical infrastructures, and human and financial resource capacity requirements in entomology and vector control operations.

## Abbreviations

ACT: artemisinin-based combination therapy
DDT: dichloro-diphenyl-trichloro ethane
DQS: data quality self-assessment
GPIRM: global plan for insecticide resistance management
IRS: indoor residual spraying
IRM: insecticide resistance management
ITN: insecticide treated nets
IVM: integrated vector management
LLINs: long-lasting insecticidal nets
LSM: larval source management
NMCP: National Malaria Control Programme
NVDCP: National Vector Borne Disease Control Programme
WHO: World Health Organization

## References

[1] Hay SI, Smith DL, Snow RW (2008). Measuring malaria endemicity from intense to interrupted transmission. Lancet Infect Dis.

[2] Guerra CA, Snow RW, Hay SI (2006). Mapping the global extent of malaria in 2005. Trends Parasitol.

[3] WHO/Global Malaria Programme (2013) World Malaria Report 2013. World Health Organization, Geneva, Switzerland. http://www.who.int/malaria/publications/world_malaria_report_2013/report/en/. Accessed 27 Jan 2015

[4] WHO/Global Malaria Programme (2007). Draft global technical strategy for malaria 2016–2030.

[5] WHO/Global Malaria Control and Elimination (2008). Report of the technical review.

[6] WHO (2007) Malaria elimination: a field manual for low and moderate endemic countries. World Health Organization, Geneva, Switzerland

[7] MoH (2014) Malaria vector control strategy 2015–2019. Toward Universal Coverage. National Malaria Control Programme Community Health Sciences Unit. Lilongwe, Malawi

[8] WHO (2004) Global strategic framework for integrated vector management. WHO/CDS/CPE/PVC/2004.10. World Health Organization, Geneva. http://whqlibdoc.who.int/hq/2004/WHO_CDS_CPE_PVC_2004_10.pdf. Accessed 23 May 2015

[9] WHO (2012) Global plan for insecticide resistance management (GPIRM); Programme WGM, editor. World Health Organization, Geneva, Switzerland

[10] WHO (2012) Disease surveillance for malaria elimination: an operational manual. World Health Organization, Geneva, Switzerland

[11] WHO (2013) Guidance on the strengthening of reporting systems on DDT in relation to disease vector control. Global Malaria Programme, World Health Organization, Geneva, Switzerland

[12] WHO (2012) Training module on malaria control: malaria entomology and vector control. World Health Organization, Geneva, Switzerland

[13] WHO (2013) Guidance on the development of a national insecticide resistance monitoring and management plans, including annual work-plans. World Health Organization, Geneva, Switzerland

[14] WHO (2013) Indoor residual spraying: an operational manual for indoor residual spraying (IRS) for malaria transmission control and elimination. World Health Organization, Geneva, Switzerland

[15] WHO (2013) Larval source management: a supplementary measure for malaria vector control: an operational manual. World Health Organization, Geneva, Switzerland

[16] WHO (2011) Guidelines for monitoring the durability of long-lasting insecticidal mosquito nets under operational conditions (WHO/HTM/NTD/WHOPES/2011.5) Geneva, World Health Organization. http://whqlibdoc.who.int/publications/2011/9789241501705_eng.pdf. Accessed 23 May 2015

[17] MoHSS (2014) National policy on malaria. Ministry of Health and Social Services. Republic of Namibia. Windhoek, Namibia

[18] MoHSS (2014) Namibia national vector control guidelines. Ministry of Health and Social Services. Republic of Namibia. Windhoek, Namibia

[19] WHO (2009) Development of a global action plan for integrated vector management (IVM). Report of a WHO Consultation. World Health Organization, Geneva. http://whqlibdoc.who.int/hq/2009/WHO_HTM_NTD_VEM_2009.1_eng.pdf. Accessed 23 May 2015

[20] MoHSS (2014) Guidelines for entomological surveillance for malaria vectors in Namibia. Ministry of Health and Social Services. Republic of Namibia. Windhoek, Namibia

[21] MoHSS (2014) Workshop on harmonization and consensus building on reporting and reducing the reliance on DDT use for malaria vector control. Ministry of Health and Social Services. Republic of Namibia. Windhoek, Namibia

[22] MoHSS (2014) Format for compilation of data on IRS implementation and coverage. Ministry of Health and Social Services. Republic of Namibia. Windhoek, Namibia

[23] Noor AM, Alegana VA, Kamwi RN, Hansford CF, Ntomwa B, Katokele S (2013). Malaria control and the intensity of *Plasmodium falciparum* transmission in Namibia 1969–1992. PLoS One.

[24] Coetzee M, Craig M, le Sueur D (2000). Distribution of African malaria mosquitoes belonging to the *Anopheles gambiae* complex. Parasitol Today.

[25] Government Republic of Namibia Ministry of Health and Social Services National Vector-borne Diseases Control Programme (2013) Malaria Annual Report 2012/2013. Ministry of Health and Social Services, Government Republic of Namibia, Windhoek

[26] Coetzee M, Knols BGJ, Louis C (2006). Malaria and dengue vector biology and control in Southern and Eastern Africa. Bridging laboratory and field research for genetic control of disease vectors.

[27] Gueye CS, Gerigk M, Newby G, Lourenco C, Uusiku P, Liu J (2014). Namibia’s path toward malaria elimination: a case study of malaria strategies and costs along the northern border. BMC Public Health.

[28] Ntomwa BN, Usuku P, Govere JN, Manga L, Koekemoer LL, Hunt RH (2006). Distribution of members of the *Anopheles gambiae* Giles s.l. complex in Namibia and susceptibility to insecticides used for malaria control. Afr Entomol.

[29] MoHSS (2014) Reports on the susceptibility and wall bio-assay tests for the evaluation of national malaria control program (NMCP) 2009–2013 transmission seasons. Ministry of Health and Social Services. Republic of Namibia. Windhoek, Namibia

[30] Government Republic of Namibia Ministry of Health and Social Services National Vector-borne Diseases Programme (2010) Malaria Strategic Plan 2010–2016. Windhoek

[31] Hansford CF (1990) Malaria Control in Namibia. National Institute for Tropical Disease. Tzaneen, South Africa: Department of National Health and Population Development (unpublished document)

[32] Snow RW, Amratia P, Kabaria CW, Noor AM, Marsh K (2012). The changing limits and incidence of malaria in Africa: 1939–2009. Adv Parasitol.

[33] Mabaso M, Sharp B, Lengeler C (2004). Historical review of malarial control in southern African with emphasis on the use of indoor residual house-spraying. Trop Med Int Health.

[34] Government Republic of Namibia Ministry of Health and Social Services (2014) Malaria indicator survey in Namibia. Republic of Namibia. Windhoek, Namibia

[35] MoHSS (2014) National insecticide resistance monitoring and management plan. Ministry of Health and Social Services. Republic of Namibia. Windhoek, Namibia

[36] World Health Organization (2010) Introduction to the project on: “Establishment of efficient and effective data collection and reporting procedures for evaluating the continued need of DDT for disease vector control”, January 2011 to December 2013. Project number: GFL/3349. Global Environmental Programme

[37] Janeira F, Vicente JL, Kanganje Y, Moreno M, Do Rosario VE, Cravo P (2008). A primer-introduced restriction analysis-polymerase chain reaction method to detect knockdown resistance mutations in *Anopheles gambiae*. J Med Entomol.

[38] Thomsen EK, Strode C, Hemmings K, Hughes AJ, Chanda E, Musapa M (2014). Underpinning vector control through informed insecticide resistance management. PLoS One.

[39] Chanda E, Hemingway J, Kleinschmidt I, Reman A, Ramdeen A, Phiri FN (2011). Insecticide resistance and the future of malaria control in Zambia. PLoS One.

[40] Munhenga G, Masendu HT, Brooke BD, Hunt RH, Koekemoer LK (2008). Pyrethroid resistance in the major malaria vector *Anopheles arabiensis* from Gwave, a malaria-endemic area in Zimbabwe. Malar J.

[41] Mouatcho JC, Munhenga G, Hargreaves K, Brooke BD, Coetzee M, Koekemoer LL (2009). Pyrethroid resistance in a major African malaria vector *Anopheles arabiensis* from Mamfene, northern KwaZulu-Natal, South Africa. S Afr J Sci.

[42] Hargreaves K, Hunt RH, Brooke BD, Mthembu J, Weeto MM, Awolola TS (2003). *Anopheles arabiensis* and *An. quadriannulatus* resistance to DDT in South Africa. Med Vet Entomol.

[43] Hemingway J, Vontas J, Poupardin R, Raman J, Lines J, Schwabe C (2013). Country-level operational implementation of the global plan for insecticide resistance management. Proc Natl Acad Sci USA.

[44] Chanda E, Masaninga F, Coleman M, Sikaala C, Katebe C, MacDonald M (2008). Integrated vector management: the Zambian experience. Malar J.

[45] World Health Organization Regional Office for Africa (2011) WHO Namibia and Angola welcomes historical cross border agreement to close the net on malaria. Ondjiva, 2011. http://www.afro.who.int/en/media-centre/pressreleases/item/2921-who-namibia-and-angola-welcomes-historical-cross-border-agreement-to-close-the-net-on-malaria.html. Accessed 05 Feb 2014

[46] Roll Back Malaria (2012) Annual trans-kunene malaria initiative (TKMI). Stakeholders Meeting. Gaborone, Botswana. http://www.rbm.who.int/countryaction/docs/sarn/TKMIstakeholdersMeeting2012.pdf. Accessed 05 Feb 2014

[47] Kamwi RN (2005) Malaria situation in Namibia. A study of vector species and effectiveness of the past and current control strategies in selected parts of Namibia. Ph.D. thesis, University of Namibia

